# Radiation synthesis and chemical modifications of p(AAm*-co-*AAc) hydrogel for improving their adsorptive removal of metal ions from polluted water

**DOI:** 10.1038/s41598-023-49009-0

**Published:** 2023-12-11

**Authors:** Israa Kamal Abdel Maksoud, Ghada Bassioni, Norhan Nady, Sherif A. Younis, Mohamed Mohamady Ghobashy, M. S. A. Abdel-Mottaleb

**Affiliations:** 1 Department of Chemistry, Faculty of Science, Ain Shams University, Abbassia, Cairo, 11566 Egypt; 2 Department of Physics and Engineering Mathematics, Faculty of Engineering, Ain Shams University, Cairo, Egypt; 3 Polymeric Materials Research Department, Advanced Technology and New Materials Research Institute, City of Scientific Research and Technological Applications (SRTA-City), New Borg El-Arab City, Alexandria, 21934 Egypt; 4 Analysis and Evaluation Department, Egyptian Petroleum Research Institute (EPRI), Nasr City, Cairo, 11727 Egypt; 5 Radiation Research of Polymer Department. National Center for Radiation Research and Technology (NCRRT), Egyptian Atomic Energy Authority (EAEA), Cairo, Egypt

**Keywords:** Environmental sciences, Chemistry, Materials science

## Abstract

The research focuses on utilizing gamma irradiation to synthesize polyacrylic acid-co-polyacrylamide p(AAm-co-AAc) hydrogels. The effect of synthetic parameters on physicochemical features of p(AAm-co-AAc) hydrogls were examined, including acrylic acid (AAc): acrylamide (AAm) weight ratios, monomer concentration, and gamma irradiation dosage (kGy). At the optimum synthetic conditions (30 kGy and 75% AAc), different chemical modifications are explored to incorporate sulfonate, hydroxyl, carboxyl, cysteine, thiol, and amine functional groups within the bare hydrogel (Cpd 0) structure. Fourier-transform infrared spectroscopy (FTIR) and scanning electron microscopy (SEM) analyses confirmed the success development of functionalized hydrogels (namely Cpd 1 to 6) with three-dimensional porous structures. These modified hydrogels include Cpd 1, a sulfonated hydrogel through a sulfonation reaction; Cpd 2, modified via NaOH hydrolysis; Cpd 3, modified using thionyl chloride; Cpd 4, incorporating cysteine modification through reaction with cysteine; Cpd 5, with 4-(Dimethylamino) benzaldehyde; and Cpd 6, modified with 3,4-Dimethylbenzoic acid.The effect of hydrogel composition and surface functionalities on the swelling capacity and interactions with scale-forming/heavy metal ions (e.g., Ba^2+^, Sr^2+^, and Cu^2+^) was investigated in saline water solution (NaCl = 1000 mg/L). Batch adsorption studies reveal that all modified hydrogels exhibited higher removal efficiency for the three metal ions than unmodified p(AAm-co-AAc) hydrogel, validating the key role of surface functionalities in tailoring hydrogel affinity for metal ions adsorption. Amongst these, NaOH-treated hydrogel (Cpd 2) outperformed all other modified ones in the removal of Cu^2+^, Ba^2+^, and Sr^2+^ ions, with maximum capacities of 13.67, 36.4, and 27.31 mg/g, respectively. Based on adsorption isotherm and kinetic modeling, the adsorption process of the three metal ions onto all modified hydrogels better obeyed Freundlich isotherm and pseudo-first-order kinetic models. Thermodynamic studies also indicated that the adsorption behavior of Sr^2+^ ions can exhibit both exothermic and endothermic characteristics, depending on the nature of hydrogel surface chemistry. Conversely, the adsorption process of Cu^2+^ and Ba^2+^ ions onto all modified hydrogels is endothermic, suggesting favorable chemical adsorption mechanisms. These findings reveal that the specific adsorption performance of hydrogel is dependent on the type of modification and the targeted heavy metal ions. Based on the nature of hydrogel surface functionality, surface modifications can change the charge density, hydrophilicity, and overall chemical environment of the hydrogel, offering a versatile approach to optimize the adsorption affinity/selectivity of hydrogel's in removing scale-forming/heavy metals from water solutions.

## Introduction

The growth of industries has played a vital role in advancing society, improving living standards, and driving economic development^1^. However, this industrial progress has come at a cost with the increased pollution of water bodies that poses significant risks to the environment and living organisms^2^. In particular, releasing heavy metal ions into water sources has become a considerable concern and a pressing issue that requires immediate attention^3^. In particular, copper (Cu^2+^), barium (Ba^2+^), and strontium (Sr^2+^) ions are among the toxic heavy metals on human health and the environment. The World Health Organization (WHO) has established maximum permissible limits for these ions in drinking water at 2 mg/L for Cu^2+^, 0.7 mg/L for Ba^2+^, and 1.5 mg/L for Sr^2+^^4^. Copper contamination in aquatic ecosystems can adversely affect marine organisms, disrupting their physiological processes and overall ecosystem balance^5^. The toxicity of barium primarily depends on its cumulative health effects, including kidney damage and neurological disorders^6^. Strontium is a naturally occurring element in rocks, soil, and water. While strontium is not highly toxic, specific forms, like radioactive strontium-90, can pose serious health risks. Radioactive strontium-90 can enter the food chain through contaminated water and soil, leading to increased risks of cancer, particularly bone cancer^7^. These toxic metal ions can be found in various sources like pesticides, fungicides, refineries, fertilizers, mining activities, smoking, nuclear fission plants, chemical industries, paints, electroplating, welding, automobiles, batteries, and many others^8^. Their contamination of water bodies can harm aquatic ecosystems and pose serious health risks to humans^9^.

Over the past decades, numerous research efforts have been devoted to developing efficient methods for removing toxic ions from water to address this challenge in recent years. Among the various approaches available, adsorption has emerged as a promising technique due to its effectiveness, scalability, and versatility^10^. Adsorption involves the attachment of pollutants to the surface of a solid material, known as an adsorbent, thereby removing them from the water^11^. For instance, different adsorbent materials (e.g., polymeric, carbon, metal oxides, hybrid metal–organic composite, etc.) have been prepared and proposed for heavy/toxic metal ion adsorption. Amongst, polymeric hydrogels have recently emerged as promising adsorbent materials for removing heavy/poisonous metal ions from water due to their unique properties, including high porosity and hydrophilicity^12^. Specifically, polymeric hydrogels with 3D networking structures have gained considerable attention for effectively removing metal ions from different types of wastewater^13^. The 3D polymeric hydrogels are formed through crosslinking hydrophilic polymers, resulting in a network with high porosity, surface functionality, and capacitance to uptake water contaminants (i.e., via swelling properties). Hydrogels demonstrated multiple advantages, such as the highly hydrophilic nature with many polar surface functionalities that accelerate the formation of electrostatic and coordination bonds with heavy metal cations^14^. Hydrogels are generally non-toxic with good biocompatibility, making them suitable for environmental applications^15^.

For hydrogel synthesis, the radiation technique is recognized as a valuable method for crosslinked hydrogel^16,17^ to be used as a template^18^, semi-permeable membrane^19^, and blend polymer^20^ based on green renewable resources to protect the environment from harmful effects^21^^–^^23^. Hydrogels-based renewable biopolymers have also attracted considerable research interest for potential applications in environmental remediation, such as wastewater treatment and pollution protection^24^. Since the surface chemistry (specifically, the functional chelating entities) of the polymer matrix is primarily responsible for the hydrogel's ability to remove metal, it is also possible to modify the monomer structure during in-situ polymerization and change the surface functional moieties ex-situ using chemical modification techniques. In this respect, many efforts have been made to incorporate various surface functionalities (such as sulfonic, hydroxyl, phosphate, carboxylic acid, amine/amide, and epoxy moieties) on hydrogel adsorbents to increase their adsorption affinity for heavy metal cations through complexation and electrostatic interaction mechanisms^25,26^. For instance, acrylamide (AAm) and acrylic acid (AAc) are the most common monomers synthesizing hydrogel adsorbents to remove metal ions^27^. Using AAm and AAc monomers, graft copolymerization reaction of poly (acrylamide-co-acrylic acid) hydrogel was carried out to involve carboxyl surface functionalities for enhanced adsorption removal of some heavy metals (e.g., Cr^2+^, Cu^2+^, Ni^2+^, and Zn^2+^)^28^. By copolymerizing acrylic acid (AA) and sodium styrene sulfonate (SS), modified carboxymethyl cellulose (CMC)-based hydrogels with sulfonate groups (-SO3Na) were also created for efficient removal of Cu^2+^ and Zn^2+^ ions^29^. However, it should be noted that three crucial factors, including (i) the polymer volume fraction of hydrogel, (ii) the degree of cross-linking, and (iii) the size of the network mesh, could affect the metal capacity of radiation-induced-graft polymeric adsorbents. These variables affect the swelling ratio (the amount of absorbed water), mechanical strength, and the diffusivity of reactant molecules (heavy metal ions) into the hydrogel structure. This in turn affects the stability, toughness, and capacitance of the adsorbent during water treatment.

On the above basis, this research article aims to provide a comprehensive study of using the radiation-induced synthesis method to prepare hydrogel-based adsorbents with different functionalities to effectively remove metal ions (e.g., Cu^2+^, Ba^2+^, and Sr^2+^ ions) from water bodies. The chosen gamma irradiation will be selected to control the crosslinking polymerization of acrylamide (AAm) and acrylic acid (AAc) chains, forming a 3D of p(AAc-co-AAm) hydrogel network with high porosity and swelling capacity. Hence, the gamma radiation synthesis approach will include optimizing irradiation parameters based on the characteristics of the resulting hydrogels. The best hydrogel with the highest swelling capacitance will further be selected for different chemical modification strategies to introduce sulfonate, carboxyl, thiol, hydroxyl, and amine groups that effectively chelate and exchange metal ions from natural and low-saline water solution (at 1000 mg/L NaCl). The hydrogel chelation and ion exchange properties will also be evaluated through batch experiments as a function of operating parameters like temperature, contact time (t), initial metal ions (Cu^2+^, Ba^2+^, and Sr^2+^) concentrations, solution pH, and hydrogel dosage. Finally, the prospects in hydrogel-based adsorbents for metal ions removal will be discussed, highlighting potential research directions and emerging technologies. The novelty of this study lies in developing a hydrogel with excellent chelation and ion exchange capabilities for removing heavy and toxic metals from water, explicitly focusing on Cu^2+^, Ba^2+^, and Sr^2+^ ions. The adsorption mechanisms involved in the interaction among ions and hydrogel-based adsorbents will be examined, shedding light on the factors influencing adsorption capacity, selectivity, isotherms, thermodynamics, and kinetics.

## Experimental methods experimental

### Materials

Acrylic acid (AA) and acrylamide (AAm) monomers (99%, analytical grade) were purchased from Fluka Co., Germany. Sulfanilic acid, thionyl chloride (SOCl_2_), cysteine with 4-(Dimethylamino) benzaldehyde, and 3,4-Dimethylbenzoic acid were supplied as analytical grade chemicals (> 97% purity) from Sigma-Aldrich. Sodium hydroxide (NaOH: 98%) and Sodium chloride (NaCl: 99%) were purchased from ADWICK” commercial.

### Preparation of p(AAm*-co-*AAc) hydrogel for metal ions removal.

**Figure 1:**
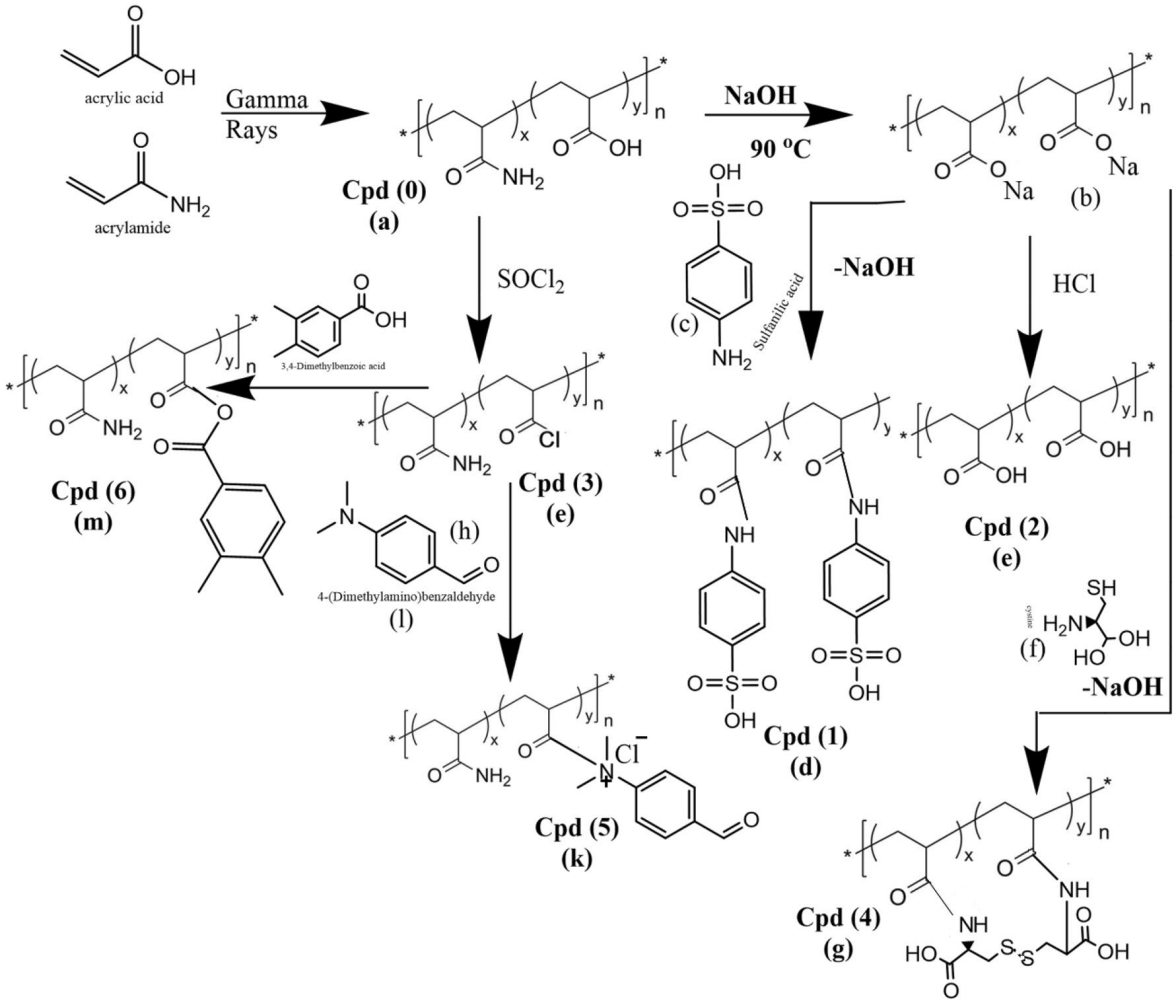
Chemical modification of p(AAm*-co-*AAc) hydrogel.

The synthesis procedure of hydrogel consisting of crosslinked polyacrylic acid and polyacrylamide networks using the gamma irradiation technique is shown in Fig. 1. In this procedure, different volume-to-weight ratios (12, 22, or 27 v/wt %) of acrylic acid (AAc: 1.25, 2.3, or 2.8 mL) and acrylamide (AAm: 3.75, 6.9, or 8.5 g) comonomers was dissolved in 40 mL of deionized (DI) water. The obtained AAc/AAm solution mixtures were subject to a gamma-ray dose of 10 kGy. Different commoner compositions of (AAc/AAm) at v/wt% of (25/75), (50/50), and (25/75) have been prepared at the gamma-ray dose of 10 kGy. Swelling and gel fraction experiments tested the prepared hydrogel samples of p(AAm*-co-*AAc) to a candidate for the best commoners compositions to study the impact of irradiation doses. The effect of irradiation doses on the swelling and gel fraction of p(AAm*-co-*AAc) was carried out at 10, 20, and 30 kGy.

### Chemical modification of p(AAm*-co-*AAc) hydrogel

Different chemical modification strategies were carried out to functionalize the bare p(AAm*-co-*AAc) hydrogel (coded as Cpd 0) with thiol (-SH), amino (-NH_2_), or carboxyl (-COOH), allowing the development of six functional hydrogels (coded as Cpd 1 to Cpd 6). These functional groups can selectively bind and remove the target ions from the solution. The chemical modification scheme of Cpd 0 to prepare modified Cpd 1 to Cpd 6 is illustrated in Fig. 1.

#### Reaction with sulfanilic acid

Dispersal 1.5 g of p(AAm*-co-*AAc) hydrogel sample (Cpd 0) is reacted with NaOH at a temperature of 90 °C. The obtained sample was dispersed in 120 mL of water containing 1.25 mol of sulfanilic acid (Fig. 1). The hydrogel sample is thoroughly mixed with the sulfanilic acid. The reaction mixture is stirred for one hour at ambient temperature, then hydrothermally treated at 60 °C for three hours. The sulfanilic acid reacts with functional groups in the hydrogel, modifying the hydrogel by introducing new groups. At the end of the modification process, the modified p(AAm*-co-*AAc) hydrogel is filtered, then washed with ethanol and 0.1 M HCl solutions to remove unreacted sulfanilic acid or reaction byproducts. Finally, the hydrogel is washed with ethyl acetate to ensure the removal of residual solvents and impurities. The washed sample of sulfonated p(AAm*-co-*AAc) hydrogel (coded as Cpd1) is dried at 50 °C, and then stored in a closed glass bottle for further use.

#### Reaction with sodium hydroxide

A certain weight of p(AAm*-co-*AAc) hydrogel sample (Cpd 0) was allowed to swell in water, then heated at 90 °C in NaOH (0.5 M) solution, followed by the reaction with 1 M HCl to give Cpd (2), as shown in Fig. 1. The approach involves alkaline hydrolysis of the amino (–NH_2_) groups in the Cpd 0 sample and conversion into carboxyl (-COOH) groups, followed by a reaction with HCl to obtain the modified hydrogel (Cpd 2).

#### Reaction with thionyl chloride

In a three-neck flask, a known weight of the bare p(AAm*-co-*AAc) hydrogel (Cpd 0, 0.5 g) is added to thionyl chloride (SOCl_2_, 2 mol) solution prepared in methylene chloride solvent. The hydrogel and SOCl_2_ solution mixture is stirred under the conditions (80 °C for 7 h). Thermal treatment facilitates the response among the hydrogel and SOCl_2_, leading to the desired modification. After the specified reaction (t), ice crystals are added to quench any unreacted SOCl_2_ and prevent further reaction. Then, the obtained modified p(AAm*-co-*AAc) hydrogel (Cpd 3; Fig. 1) is filtered and washed with methylene chloride solvent to remove any residual reactants or byproducts. Subsequently, the hydrogel is dried in an oven at 50 °C (24 h) and then stored in a closed glass container till further use.

#### Reaction with cystine

An aqueous solution of 0.8 mol of cystine (100 mL) is freshly prepared and added slowly to the Cpd 0 hydrogel (0.5 g) under stirring to ensure uniform mixing. After the complete addition of the cystine solution, the mixture is allowed to stand overnight. The mixture is then allowed for thermal soxhtelation under stirring at 50 °C for 60 min to facilitate the reaction process between cysteine and hydrogel. After the thermal treatment, the solid product of the modified p(AAm*-co-*AAc) hydrogel (termed Cpd 4, Fig. 1) is filtered off from the reaction mixture and then dried to remove any remaining moisture and solvents.

#### Reaction with 4-(Dimethylamino)benzaldehyde

A solution of 0.2 mol of 4-(Dimethylamino)benzaldehyde is prepared in 100 mL tetrahydrofuran (THF). The prepared 4-(Dimethylamino)benzaldehyde solution is added drop-wise to the modified p(AAm*-co-*AAc) hydrogel (Cpd 3, 0.5 g) with stirring to ensure proper mixing and distribution of the solution. After adding the 4-(Dimethylamino)benzaldehyde solution, the mixture can stand at ambient temperature for 24 h, followed by soxhtelation at 65°C for six hours. The elevated temperature and stirring help facilitate the desired modification reaction by the promoted interaction between the hydrogel and 4-(Dimethylamino)benzaldehyde. After the reaction, the obtained solid product is thoroughly washed with ethanol and water to remove any residual reactants or byproducts. The washed solid of the modified p(AAm*-co-*AAc) hydrogel is then dried to remove any remaining moisture and labeled as Cpd 5, according to Fig. 1**.**

#### Reaction with 3,4-Dimethylbenzoic acid

A 0.25 mol of 3,4-Dimethylbenzoic acid solution is prepared in 100 mL of acetonitrile and mixed with the Cpd 3 hydrogel sample (0.5 g) under stirring for seven hours. After the mixing step, the mixture is heated to 60 °C under stirring for six hours to promote the reaction of 3,4-Dimethylbenzoic acid with the hydrogel. After the reaction, the solid sample obtained is separated by filtration and washed with ethanol to purify the modified hydrogel. The washed solid sample is finally dried to remove any remaining moisture and labeled as Cpd 6, according to Fig. 1**.**

### Characterization

Attenuated total reflectance-Fourier transform infrared (ATR-FTIR) spectroscopy and scanning electron microscopy (SEM) characterization techniques were employed to evaluate the surface functionality and structural changes of the p(AAm*-co-*AAc) hydrogel before and after chemical modification strategies. ATR-FTIR spectroscopy was performed using a Vertex 70 FTIR spectrometer equipped with a HYPERION™ series microscope from Bruker Optik GmbH, Germany. ATR-FTIR spectroscopy is a powerful technique for providing information on the modified hydrogels' surface functionality and chemical structure. The OPUS 6.0 software from BRUKER was used for data processing, including baseline correction using the rubber band method with CO_2_ bands excluded. The effect of chemical modification methods on the morphological features and structural characteristics of p(AAm*-co-*AAc) hydrogel were also evaluated using an SEM microscope (model Zeiss-EVO, MA-10, variable UK).

### Adsorption study

This study uses a synthetic water solution with 1000 mg/L salinity (as NaCl) and a mixture of HM cations (e.g., Cu^2+^, Ba^2+^, and Sr^2+^ ions) to simulate the actual composition of natural surface water bodies. For the adsorption process, the adsorption affinity of modified p(AAm*-co-*AAc) hydrogels for the three HM ions (Cu^2+^, Ba^2+^, and Sr^2+^) from their water solutions were conducted in batch mode under fixed operating conditions (2 g/L adsorbent mass, agitation speed of 150 rpm, and solution pH 6.5 ± 0.3). At different (t)intervals (1–24 h), the effluent water sample (5 mL) was withdrawn, then filtered and subjected to analysis using Ion-chromatography (IC Dionex DX-600, USA) and atomic absorption spectroscopy (AAS: Analytik Jena Zenit 700p, Germany) to quantify the residual concentration of HM ions in the treated water samples. The adsorption affinity of hydrogels (bare and modified) for the three HM cations was also investigated and compared in relation to the effects of adsorption time (1–24 h), initial HM ions’ concentrations (Cu^2+^: 1–50 mg/L; Ba^2+^: 10 -100 mg/L; Sr^2+^: 10–100 mg/L), and solution temperature (15−45 °C). These comprehensive studies aimed  to get a deeper insight into the adsorption mechanism based on kinetic, isotherm, and thermodynamic analyses. Exact repeatability for each experimental run was also tested to calculate the systematic error during adsorption. The adsorption performance of tested hydrogel adsorbents for HM uptake from water solution was evaluated by different metrics like adsorption efficiency (RE (%), Eq. (1)) and capacity of adsorption (Q_t_ (mg/g), Eq. (2)).

1 $$RE\left(\%\right)=\frac{{C}_{o}-{C}_{t}}{{C}_{o}}\times 100$$

2 $${Q}_{t}=\frac{{C}_{o}-{C}_{t}}{m}\times V.$$

Here, C_o_ and C_t_ (mg/L) are the initial concentration and residual concentration of tested HM ions in water solution before and after the adsorption process, respectively, at different (t) intervals (t, h). The terms m (g) and V (L) refer to the hydrogel mass (gram) and solution volume (liter) used during the adsorption process, respectively.

## Results and discussion

### Characterization data

#### Effect of synthetic conditions on the swelling and gel fraction of p(AAm*-co-*AAc) hydrogel

**Figure 2:**
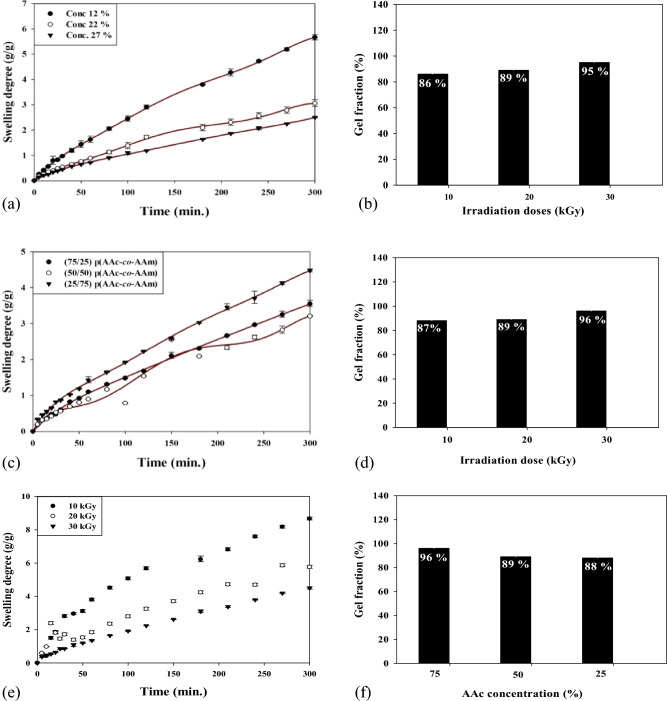
Effect of comonomer concentration and gamma irradiation on the swelling degree and gel fraction of p(AAm*-co-*AAc) hydrogel.

Figure 2a shows the p(AAm*-co-*AAc) hydrogel materials swelling degree as a function of comonomer concentration (AAc and AAm) from 12 to 27 v/wt%. At a co-monomer concentration of 12 v/wt%, the hydrogel exhibited the highest swelling degree of 400% after 240 min. As the co-monomer concentration increased to 22 v/wt% and 27 v/wt%, the swelling degree decreased to 240% and 200%, respectively. This reduction in the swelling capacity could be ascribed to the impact of higher co-monomer concentrations on crosslinking density, the hydrophilic nature of hydrogel, and the polymer network structure that played a key role in determining the swelling behavior of hydrogels. For the gel fraction in Fig. 2b, it was denoted that the p(AAm*-co-*AAc) hydrogel had the highest gel fraction at 27 v/wt % co-monomer concentration level. A higher gel fraction suggests higher crosslinking and a more stable and structurally robust hydrogel. Compared to hydrogels with higher polyacrylamide content in Fig. 2c, the lower swelling degrees of p(AAm*-co-*AAc) hydrogels at higher acrylic acid content could also attributed to intermolecular hydrogen bonds formed by acrylic acid carboxyl groups (-COOH). These intermolecular hydrogen bonds restrict the uptake of water molecules by the hydrogel, leading to a decrease in swelling capacity. In contrast, hydrogels with higher polyacrylamide content are expected to have a higher swelling degree due to the absence of carboxyl groups and the resulting lower propensity for hydrogen bond formation. Figure 2d shows that the gel fraction of the p(AAm*-co-*AAc) hydrogel increases as the acrylic acid content decreases. The gel fraction measures the extent of crosslinking within the hydrogel network. A higher gel fraction suggests higher crosslinking and a more stable and structurally robust hydrogel. The impact of gamma irradiation dose (10–30 kGy) on the degree of swelling and gel fraction of p(AAm*-co-*AAc) (25:75) hydrogel samples was studied, and the results are shown in Fig. 2e,f. As can be observed in Fig. 2e and f, t the p(AAm*-co-*AAc) (25:75) hydrogel samples demonstrated a decrease in the swelling degree and an increase in the gel fraction as gamma irradiation doses rose from 10 to 30 kGy. Gamma irradiation generates free radicals within the hydrogel matrix to initiate crosslinking reactions among the polymer chains, resulting in a denser network structure at a higher irradiation dose. This denser network restricts the uptake of water molecules, leading to a decrease in the degree of swelling.

#### Surface functionalities of p(AAm*-co-*AAc) hydrogels

**Figure 3:**
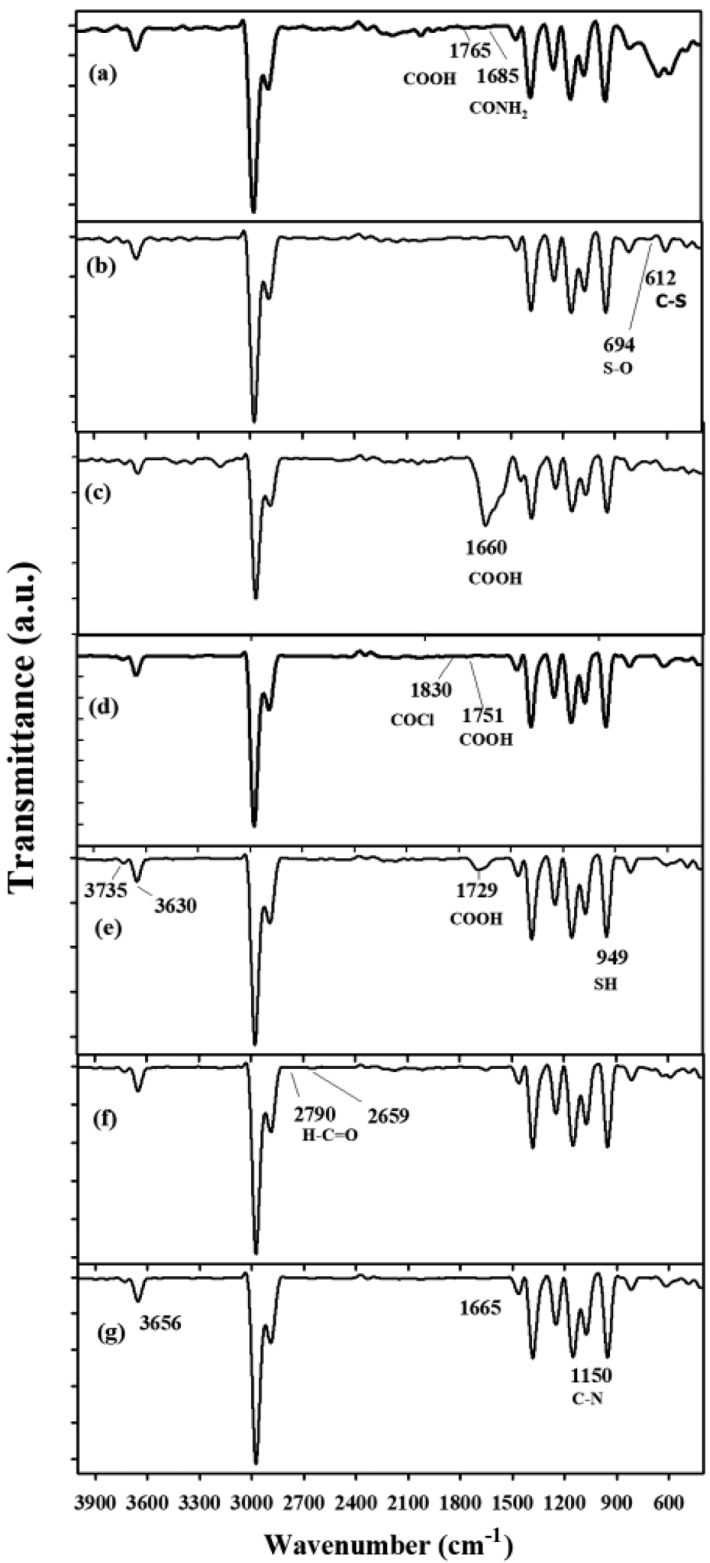
The FTIR spectra of bare and chemically modified p(AAm*-co-*AAc) hydrogels: (**a**) Cpd (0), (**b**) Cpd (1), (**c**) Cpd (2), (**d**) Cpd (3), (**e**) Cpd (4), (**f**) Cpd (5), and (**g**) Cpd (6).

Figure 3 shows the FT-IR spectra of all poly(acrylic acid*-co-*acrylamide) hydrogels (Cpd x, where x = 0 to 6) before and after modification strategies. The FTIR spectrum of the unmodified hydrogel (Cpd 0: Fig. 3a) showed absorption peaks at around 3656 cm^−1^ and 3531 cm^−1^, assigned to the stretching vibrations of the –OH groups in the hydrogel structure from AAc and AAm monomers, respectively. Two peaks at 2976 cm^−1^ and 2855 cm^−1^ are also observed, corresponding to the asymmetric and symmetric stretching vibrations of the –CH_2_– groups. The peak at 1450 cm^−1^ is assigned to the bending vibration of the –OH hydroxyl groups. The peaks at 1059 cm^−1^ and 827 cm^-1^ are attributed to the stretching vibrations of the C–O and C–C bonds, respectively. The absorption peaks at 1685 cm^−1^ and 1756 cm^−1^ are also ascribed to the C=O stretching vibrations in the amide and carboxyl groups, respectively. For the FTIR spectrum of modified Cpd1 hydrogel (Fig. 3b), different FTIR peaks are recorded as follows: 1450 cm^−1^ (C-N stretching in benzenoid rings), 612 cm^−1^ (the stretching vibrations of C-S bonds), and 694 cm^−1^ (bending vibration of SO_3_ groups). These peaks suggest the existence of sulfonate functional groups in the modified Cpd1 hydrogel. In Fig. 3c, the characteristic FTIR peak of C=O is observed as a very broad peak at 1660 cm^−1^, indicating that the carboxylic groups in the Cpd2 hydrogel have increased compared to the blank Cpd0 sample. The existence of carboxylic groups can be attributed to the modification approach or the introduction of functional groups like acrylic acid during the synthesis. Carboxylic groups are known for their ability to form hydrogen bonds and exhibit acidic properties. They can contribute to the adsorption capacity and selectivity of the hydrogel toward specific ions or molecules^30^.

 (Fig. 3d) also shows a new characteristic peak at 1830–1751 cm^−1^ related to the stretching vibration of the acyl chloride (–COCl) group after the reaction of hydrogel with thionyl chloride (SOCl_2_). The acyl chloride functional group (–COCl) is highly reactive and can participate in various chemical reactions. The acyl chloride group can be a reactive site for further functionalization or chemical reactions. It can be utilized to introduce specific functional groups or linkers that enhance the adsorption properties or enable targeted interactions of the hydrogel with specific ions, molecules, or substances^31^. For instance, a new absorption peak  at 1729 cm^−1^ is observed in the FTIR spectrum of Cpd4 hydrogel (Fig. 3e), attributing to a carbonyl stretch vibration of (C = O) groups^32^. Further changes were observed in the broad FTIR peaks around 3735 cm^−1^ and 3630 cm^−1^ for –OH and –NH_2_ groups, respectively. A new FTIR peak of the SH bend is also located around 949 cm^−1^^33^. The FTIR spectrum of the Cpd5 (Fig. 3f) presents two characteristic related to the presence of 4-amino benzaldehyde at 2790 and 2659 cm^−1^ for the H–C=O stretching vibration of carbonyl-containing functionality and aldehydic C–H stretching vibration, respectively. In Fig. 3g, the FTIR analysis of Cpd6 hydrogel also displays the stretching vibration peaks of O–H and C=O groups at 3656 cm^-1^ and 1665 cm^−1^, respectively. The FTIR peak at 1150 cm^−1^ is also assigned to the aromatic C-N stretching, possibly originating from 4-aminobenzoic acid or other aromatic moieties introduced during the modification approach^34^. These peakssuggest incorporating 4-aminobenzoic acid, which contains hydroxyl and carboxylic acid functional groups, into the modified hydrogel. Introducing these functional groups can enhance the hydrogel's interactions with target  metal ions or molecules, providing specific adsorption or binding capabilities. The above FTIR spectra confirm the polymeric chains' microstructural configuration and chemical modifications.

#### Morphological features of p(AAm*-co-*AAc) hydrogels

**Figure 4:**
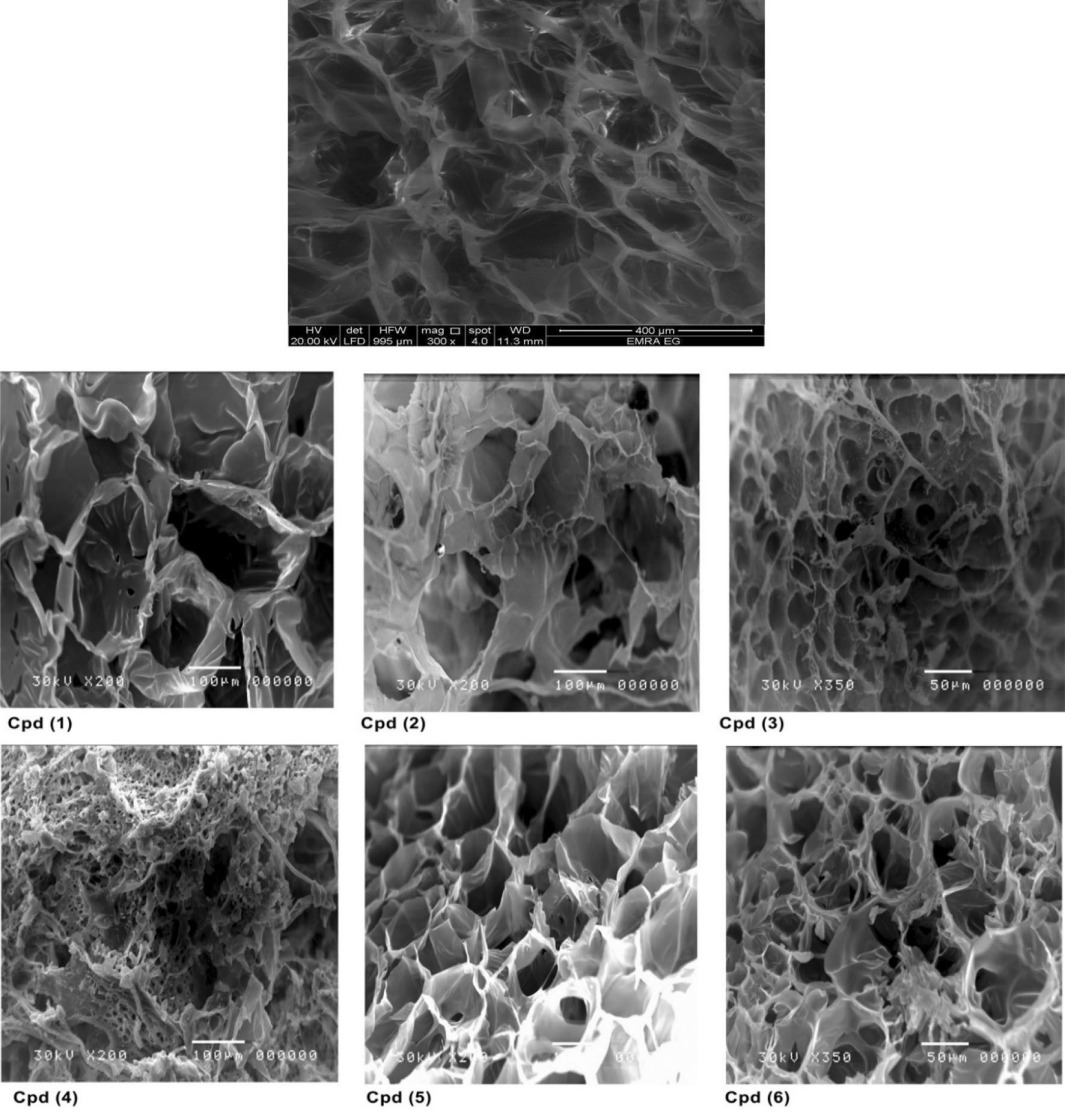
SEM images of unmodified p(AAm*-co-*AAc) hydrogel (Cpd0) and their modified forms (Cpd1 –Cpd6).

The microstructure and surface morphology of the bare and modified hydrogel samples using SEM analysis are shown in Fig. 4. The SEM images confirm that all modified p(AAm*-co-*AAc) hydrogels (Cpd 1 - Cpd6) retain porous and interconnected structures similar to the bare hydrogel sample (Cpd0). This suggests that the chemical modification approaches did not significantly alter the crosslinking structure of Cpd0 hydrogel. In particular, the Cpd0 exhibited a smooth interface with uniformLy distributed open pores. These inherent porous structures are slightly altered after chemical modification processes, confirming the successful deposition of the chemical functional groups within the prepared hydrogels. For example, the modified cpd1 hydrogel with sulfanilic acid (–SO_3_H) groups showed a higher swelling property and larger pore diameter than bare Cpd0. This is attributed to the hydrophilic nature of the sulfonate groups, which promote water absorption and swelling of the hydrogel. Compared to Cpd0, the modified Cpd4 hydrogel with cysteine functionality showed smaller pore diameters with higher agglomerates of polymer chains. Such observation was ascribed to the formation of intramolecular hydrogen bonds among the hydroxyl (OH), thiol (SH), and amino (NH_2_) groups, resulting in a denser network structure. In contrast, hydrogels with weaker intramolecular hydrogen bonding, like Cpd2, Cpd3, Cpd5, and Cpd6, showed larger pore sizes with a lower aggregation of polymer chains.

### Adsorption applicability of (AAm*-co-*AAc) hydrogels for Cu^2+^, Ba^2+^, and Sr^2+^ ions

Herein, the adsorption efficiency of blank and modified p(AAm*-co-*AAc) hydrogels for the three HMs (Cu^2+^, Ba^2+^, and Sr^2+^ ions) was assessed based on adsorption isotherm, adsorption kinetic, and thermodynamic analyses.

#### Effect of initial HM ion concentrations: adsorption isotherm modeling

**Figure 5:**
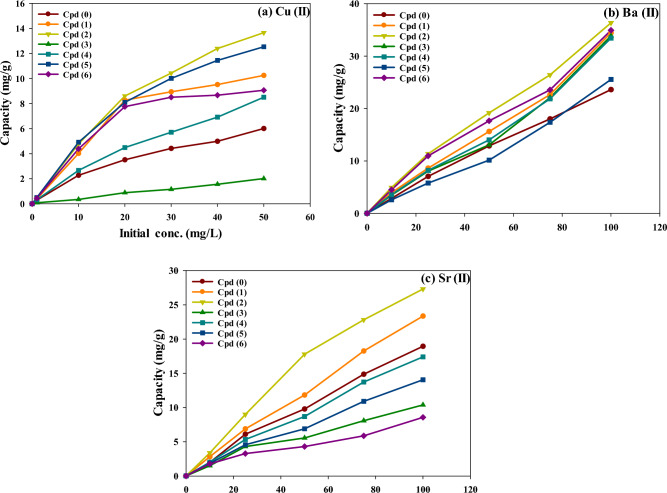
The adsorption capacity of blank and modified p(AAm*-co-*AAc) hydrogels towards varying initial concentrations of (**a**) Cu^2+^, (**b**) Ba^2+^, and (**c**) Sr^2+^ ions from synthetic water solution at 1000 mg/L salinity and 25 °C.

Figure 5 presents the adsorption capacity of the blank and modified p(AAm*-co-*AAc) hydrogel adsorbents for the three HM ions (Cu^2+^, Ba^2+^, and Sr^2+^) as a function of their varying initial concentrations. As can be seen, the adsorption capacities of all tested hydrogel adsorbents increased linearly with the initial concentration of the HM ions. Besides, all modified hydrogel samples demonstrated a higher adsorption affinity for the three HM ions than the blank sample (Cpd0). Among the modified hydrogels, the Cpd2 hydrogel adjusted with sodium hydroxide exhibited the highest affinity for the adsorption recovery of the three HM ions. Conversely, the modification strategy of hydrogel with thionyl chloride (Cpd3)significantly reduced the adsorption capability for Cu^2+^ ion removal (Fig. 5a). In comparison, the chemically modified Cpd6 hydrogel exhibited a slightly lower adsorption uptake for Ba^2+^ and Sr^2+^ ions compared to all other modified hydrogels((Fig. 5b,c).

For Cu^2+^ ion removal (Fig. 5a), the adsorption capacity of the hydrogels ranked as Cpd2 > Cpd5 > Cpd1 > Cpd6 > Cpd4 > Cpd0 > Cpd3. Specifically, the adsorption capacity of Cu^2+^ onto Cpd2 hydrogel was 2.3-fold higher than the unmodified Cpd0 hydrogel. As the initial Cu^2+^ ion concentration increased from 1 to 50 mg/L, the adsorption capacity of Cu^2+^ onto Cpd2 linearly increased from 0.50 to 13.66 mg/g, respectively. Similarly, in Fig. 5b, all modified hydrogel samples demonstrated high adsorption affinity for Ba^2+^ ions (100 mg/L) compared to the Cpd0, showing the maximum capacity rank order of Cpd2 (36.4 mg/g) > Cpd6 (34.9 mg/g) ≥ Cpd1 (34.5 mg/g) ≥ Cpd3 (33.9 mg/g) ≥ Cpd4 (33.4 mg/g) > Cpd5 (25.5 mg/g) > Cpd0 (23.6 mg). Comparatively, In the case of Sr^2+^ ions (Fig. 5c**)**, only Cpd1 and Cpd2 hydrogels exhibited a higher adsorption capacity for Sr^2+^ ions than the blank hydrogel (Cpd0) by 1.23 to 1.44 times of magnitude, respectively. All other modified hydrogels (e.g., thionyl chloride, cysteine, 4-(Dimethylamino) benzaldehyde, or 3,4-Dimethylbenzoic acid) showed a decline in the adsorption affinity for Sr^2+^ ion uptake compared to the unmodified Cpd0 adsorbent. The adsorption capacities of Ba^2+^/Sr^2+^ ions onto Cpd2 were also increased from 4.9/3.36 mg/g to 36.4/27.31 mg/g as their initial concentration levels increased from 10 to 100 mg/L, respectively. Based on the maximum adsorption capacity, the adsorption selectivity of Cpd2 (as the best performer adsorbent) for the three HM ions can be ranked as Ba^2+^ (36.4 mg/g) > Sr^2+^ (27.31 mg/g) > Cu^2+^ (13.7 mg/g) (Fig. 5a–c). These results demonstrated the effectiveness of the alkaline (NaOH) hydrolysis method in improving the adsorption property of the p(AAm*-co-*AAc) hydrogel for Cu^2+^, Ba^2+^, and Sr^2+^ ions from the salty water solution. The choice of modifying agent and the specific HM ion being targeted also played a crucial role in determining the adsorption performance of the hydrogel adsorbents.

Based on the above adsorption data, three adsorption isotherm models were employed to describe the effect of hydrogel surface functionalities on the adsorption mechanism of the adsorbate ions in an aqueous solution. Using nonlinear regression analysis, this work utilized Freundlich (Eq. (3)), Langmuir (Eq. (4)), and Tempkin (Eq. (5)) isotherm models to analyze the adsorption equilibrium mechanism of the three metal ion adsorbates by hydrogel adsorbents^22^. The Freundlich model is the earliest known empirical equation and is shown to be consistent with an exponential distribution of active centers characteristic of heterogeneous surfaces. The Langmuir equation validates monolayer adsorption onto the adsorbent surface with a finite number of identical sites. Tempkin isotherm considered the effects of indirect adsorbent/adsorbate interactions on adsorption, assuming that the heat of adsorption of all the molecules in the layer would decrease linearly with coverage due to adsorbent/adsorbate interactions 35. The comprehensive details outlining the theoretical hypothesis for the isotherm models can be found in the Supplementary Information.  

3 $${q}_{e =}{K}_{f}{C}_{e}^{1/n}$$

4 $${q}_{e}= \frac{{q}_{m}{K}_{L}{C}_{e}}{1+{K}_{L}{C}_{e}}$$

5 $${q}_{e}=\frac{RT}{{B}_{T}}ln{A}_{T}{C}_{e}$$

where q_e_ is the equilibrium capacity of adsorbed metal ions by the hydrogel (mg/g), ${q}_{m}$ is the theoretical monolayer adsorption capacity (mg/g) at equilibrium, C_e_ (mg/L) is the equilibrium concentration of the adsorbate ions. K_F_ (L/g) is the Freundlich constant related to the adsorption capacity, whereas *n*_*f*_ refers to adsorption intensity. Langmuir constant,* K*_*L*_, represents the adsorption equilibrium (L/mg). R is the universal gas constant (0.08314 kJ/mol K), and T is the adsorption temperature in Kelvin (K). Besides, B_T_ and A_T_ refer to adsorption energy (i.e., heat of adsorption: kJ/mol) and equilibrium binding constant (L/g), respectively.

According to the *R*^*2*^ values in Table 1, the adsorption equilibrium data of the three metal ions onto all hydrogels demonstrated a good fit with the three isotherm models. Amongst, the Freundlich model best describes the adsorption behavior of the three HM ions onto blank and modified hydrogels, with fitting order of Freundlich (R^2^ = 0.90–0.992), Langmuir (R^2^ = 0.77–0.991), and Tempkin (R^2^ = 0.74–0.993). The good-of-fit to the Langmuir and Freundlich models indicates that the adsorption mechanism of HM ions onto all modified hydrogels is a complex phenomenon and might follow both monolayer and multilayer adsorption mechanisms onto heterogeneous adsorbent surfaces (i.e., neglect indirect interactions). The multilayer adsorption phenomenon observed in the Freundlich model suggests that multiple sites are available for adsorption on the hydrogel surface, allowing for the formation of numerous adsorbate molecules^27^. This behavior can be attributed to the hydrogel's porous structure and heterogeneous nature, which provides ample surface area and various binding sites for the adsorbate ions^22^.

**Table 1:** Adsorption isotherm model constants of three metal ions adsorbed by blank and modified p(AAm*-co-*AAc) hydrogel adsorbents.

Hydrogels/Constants	Metal adsorbates								
	Cu^2+^			Ba^2+^			Sr^2+^		
(a)	Freundlich model								
	K_F_	n_f_	*R*^*2*^	K_F_	n_f_	*R*^*2*^	K_F_	n_f_	*R*^*2*^
Cpd (0)	0.906	1.941	0.992	1.003	1.259	0.998	0.596	1.193	0.988
Cpd (1)	3.871	3.253	0.909	0.931	0.999	0.900	0.798	1.177	0.994
Cpd (2)	5.683	3.488	0.976	6.666	2.084	0.941	3.105	1.719	0.947
Cpd (3)	0.045	1.008	0.99	0.025	0.492	0.907	0.483	1.438	0.969
Cpd (4)	1.018	1.669	0.993	0.386	0.816	0.918	0.457	1.147	0.991
Cpd (5)	5.793	4.139	0.969	0.085	0.688	0.963	0.372	1.181	0.986
Cpd (6)	4.076	3.894	0.918	4.592	1.831	0.907	0.384	1.463	0.939

(b)	Langmuir model								
Constants	q_m_	K_L_	*R*^*2*^	q_m_	K_L_	*R*^*2*^	q_m_	K_L_	*R*^*2*^
Cpd (0)	8.104	0.061	0.986	7.246	0.091	0.996	73.58	0.006	0.987
Cpd (1)	10.894	0.452	0.937	3.74	4.46	0.89	106.721	0.005	0.991
Cpd (2)	13.057	0.916	0.961	49.507	0.069	0.875	41.82	0.042	0.981
Cpd (3)	25.653	0.051	0.991	3.16	1.33	0.9	21.663	0.011	0.956
Cpd (4)	13.651	0.043	0.985	6.27	1.76	0.77	91.875	0.004	0.989
Cpd (5)	10.922	3.798	0.917	6.43	0.00715	0.931	69.196	0.003	0.981
Cpd (6)	9.455	0.757	0.992	43.68	0.065	0.843	19.953	0.008	0.904

(c)	Tempkin model								
Constants	B_T_	A_T_	*R*^*2*^	B_T_	A_T_	*R*^*2*^	B_T_	A_T_	*R*^*2*^
Cpd (0)	2.097	2.195	0.934	0.311	0.267	0.943	0.359	0.193	0.945
Cpd (1)	1.200	5.901	0.922	0.266	0.506	0.758	0.315	0.258	0.914
Cpd (2)	1.349	46.815	0.979	0.457	7.543	0.821	0.268	0.421	0.993
Cpd (3)	5.781	0.781	0.744	0.250	0.349	0.688	0.744	0.205	0.93
Cpd (4)	1.480	2.103	0.896	0.254	0.373	0.74	0.401	0.188	0.921
Cpd (5)	1.707	133.304	0.983	0.289	0.202	0.772	0.525	0.19	0.903
Cpd (6)	1.557	13.74	0.961	0.343	1.523	0.836	1.074	0.257	0.838

From the isotherm constants in Table 1**,** both Cpd2 and Cpd5 hydrogels had the highest Freundlich constant (K_F_ = 5.68–5.79 L/g) for Cu^2+^ ions. Also, Cpd2 recorded the highest K_f_ value of 6.666 and 3.105 L/g for Ba^2+^ and Sr^2+^ adsorption, respectively. Based on the K_f_ values, the adsorption selectivity of Cpd2 for the three metal ions ranked as Ba^2+^ > Cu^2+^ > Sr^2+^, agreeing with the experimental capacity order (Fig. 5). A more prominent Freundlich exponent (n_f_ > 1) also indicated more surface heterogeneity, resulting in a non-linearity adsorption isotherm. The higher surface heterogeneity is ascribed to multiple surface functional groups as verified by FTIR analysis earlier. It is also well known that the adsorption energy could provide crucial information about the strength of the adsorption interaction between metal ions and hydrogel surface functionalities. In this respect, the Tempkin B_T_ values in Table 1 represent the heat of adsorption. By considering the positive B_T_ values, the adsorption process of HM ions onto the hydrogel adsorbents could imply favorable endothermic adsorption. Analyzing the B_T_ data in Table 1, we can observe variations in the adsorption energies among the different samples and metal ions. In particular, the largest adsorption energy was observed in the presence of Cu^2+^ ions uptake, ranging from 1.20 kJ/mol (Cpd1) to 5.781 kJ/mol (Cpd3). Also, all modified hydrogel adsorbents need higher energy for Cu^2+^ adsorption than Sr^2+^ and Ba^2+^ ions. This explains the lower adsorption capacity of copper ions onto all modified hydrogels compared to barium and strontium ions from water solution. The adsorption energy of metal ions onto all hydrogel adsorbents ranked in the ascending order Ba^2+^ (K_T_ = 0.25 to 0.457 kJ/mol) < Sr^2+^ (K_T_ = 0.268 to 1.074 kJ/mol) < Cu^2+^(K_T_ = 1.20 to 5.781 kJ/mol). The lower adsorption energy of Ba^2+^ explains the reasons for the favorable Ba^2+^ uptake by blank and modified hydrogels. In this case, it is assumed that the adsorption process of Ba^2+^ ions obeyed mainly a physical adsorption mechanism (weak interaction) and can be spontaneously stimulated via relatively weaker chelating bonds under normal operating conditions. In contrast, the more considerable adsorption energies for Cu^2+^ ions declared that higher heat energy is needed to induce the chemical interaction between hydrogel surface functionalities with Cu^2+^ ions. Overall, the estimated Tempkin K_T_ values (adsorption energy constants) in Table 1 suggested that there is a significant variation in the specific interaction mechanisms among the metal ions and the hydrogel adsorbents due to the differences in the composition and structure of the p(AAm*-co-*AAc) hydrogel samples.

#### Effect of adsorption time: kinetic modeling

**Figure 6:**
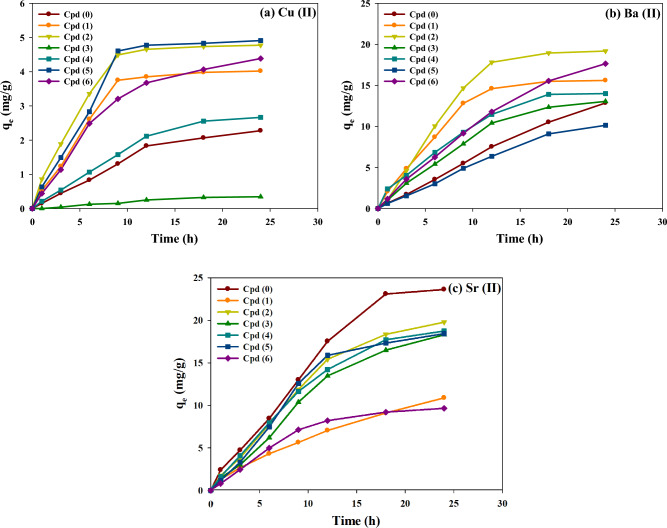
The correlation between adsorption time and adsorption capacity of three metal ions by modified and blank p(AAm*-co-*AAc) hydrogels.

Figure 6 shows the adsorption removal of three metal ions (10 mg/L Cu^2+^, 50 mg/L Ba^2+^, and 50 mg/L Sr^2+^) from a solution mixture onto all prepared p(AAm*-co-*AAc) hydrogel adsorbents as a function of adsorption time. As can be seen, there is a linear increment in the removal efficiency of the three ions during the first two hours by all modified hydrogel adsorbents, suggesting their fast initial uptake rates of metal ions. Specifically, all modified p(AAm*-co-*AAc) hydrogels exhibited a quicker removal rate for Cu^2+^ and Ba^2+^ ions than the blank Cpd0, approaching the equilibrium state after approximately 10 h. On the other hand, the equilibrium time of Sr^2+^ ion onto all hydrogels reached after around 18 h. This verifies the synergistic impact of exchangeable functional groups on the enhanced metal adsorption rate. Besides, the adsorption of Sr^2+^ ions onto the hydrogel adsorbents is slower than Cu^2+^ and Ba^2+^ ions. In particular, Cpd2 outperformed all other hydrogels for the adsorption removal of the three metal ions, following the order of Ba^2+^ (Q_e_ = 19.18 mg/g) > Sr^2+^ (Q_e_ = 17.79mg/g) > Cu^2+^ (Q_e_ = 4.78 mg/g).

Three kinetic models were applied to further investigate the obtained adsorption data, including nonlinear Lagergren’s pseudo-first-order (Eq. (6)), pseudo-second-order (Eq. (7)), and Elovich's (Eq. (8)) kinetic models^36^. The comprehensive details outlining the theoretical hypothesis for the kinetic models can be found in the Supplementary Information.

6 $${q}_{t}={q}_{e}(1-{e}^{-{K}_{1}t})$$

7 $${q}_{t}= \frac{{q}_{e}^{2}{K}_{2}t}{{1+{q}_{e}K}_{2}t}$$

8 $${q}_{t}= \frac{1}{\beta }\mathrm{ln}\left(1+\beta \alpha t\right)$$

where, q_e_ (mg/g) and q_t_ (mg/g) are the ions adsorbed at equilibrium and time (t), respectively. k_1_ (h^−1^) and k_2_ (g/mg.h) are the pseudo-first and pseudo-second rate constants, respectively. For Elovich model, α and β are the initial adsorption rate (mg/g.h) and desorption rate (g/mg) constants, respectively.

From the kinetic rate constants and fitting data (R^2^ values) in Table 2, it can be noted that the kinetic data of all metal ions onto the blank and modified hydrogels fitted well with a pseudo-first-order model (R^2^ > 0.951), followed by the pseudo-second-order model (R^2^ > 0.927) and Elovich model (R^2^ > 0.901). The high fitting adequacy of pseudo-first-order kinetic suggests that the physical adsorption (weak chemical adsorption and diffusion mechanisms) is the dominant rate-limiting step in the adsorption approach of the three metal ions onto the modified hydrogel adsorbents. Besides, the adsorption process could involve a rapid surface adsorption step, followed by slower mass transfer or diffusion processes at the equilibrium stage. Among the hydrogel samples, Cpd2 demonstrated the highest k_1_ values for Cu^2+^ (0.194 h^–1^) and Sr^2+^ (0.111 h^–1^), whereas Cpd1 was superior for the enhanced adsorption kinetic of Ba^2+^ (0.137 h^–1^). Comparatively, the lowest adsorption kinetic rates for metal ions were observed by Cpd0 for Ba^2+^ ions (k_1_ = 0.0.019 h^–1^) and Cpd3 for Cu^2+^ (k_1_ = 0.037 h^–1^) and Sr^2+^ (k_1_ = 0.0.055 h^–1^).

**Table 2:** Kinetic model constants of adsorbed metal ions onto blank and modified p(AAm*-co-*AAc) hydrogels.

Adsorbates	Cu^2+^			Ba^2+^			Sr^2+^		
(a)	Pseudo-first-order kinetic								
Hydrogels/Constants	q_e_	k_1_	*R*^*2*^	q_e_	k_1_	*R*^*2*^	q_e_	k_1_	*R*^*2*^
Cpd (0)	2.943	0.066	0.981	36.091	0.019	0.997	13.154	0.063	0.983
Cpd (1)	4.291	0.163	0.962	16.9	0.137	0.981	14.563	0.081	0.967
Cpd (2)	4.986	0.194	0.98	21.648	0.113	0.971	19.476	0.111	0.995
Cpd (3)	0.614	0.037	0.968	15.948	0.078	0.99	7.63	0.055	0.994
Cpd (4)	3.404	0.071	0.986	15.803	0.102	0.987	10.799	0.068	0.999
Cpd (5)	5.298	0.153	0.951	19.536	0.032	0.992	7.842	0.097	0.987
Cpd (6)	4.68	0.12	0.991	25.659	0.05	0.998	5.965	0.057	0.994

(b)	Pseudo-second-order kinetic								
Hydrogels/Constants	q_e_	k_2_	*R*^*2*^	q_e_	k_2_	*R*^*2*^	q_e_	k_2_	*R*^*2*^
Cpd (0)	4.597	0.010	0.977	66.627	0.0002	0.997	20.796	0.002	0.98
Cpd (1)	5.577	0.027	0.937	22.555	0.0050	0.966	22.093	0.003	0.96
Cpd (2)	6.221	0.031	0.954	30.461	0.0030	0.957	26.927	0.003	0.989
Cpd (3)	1.073	0.020	0.966	23.918	0.0020	0.986	11.993	0.003	0.993
Cpd (4)	5.236	0.009	0.982	22	0.0040	0.984	16.44	0.003	0.998
Cpd (5)	6.977	0.019	0.927	34.295	0.0010	0.991	11.304	0.006	0.98
Cpd (6)	6.417	0.015	0.983	41.499	0.0008	0.997	9.587	0.004	0.993

(c)	Elovich kinetic								
Hydrogels/Constants	α	β	*R*^*2*^	α	β	*R*^*2*^	α	β	*R*^*2*^
Cpd (0)	0.214	0.573	0.973	0.679	0.033	0.997	0.896	0.125	0.976
Cpd (1)	1.045	0.628	0.908	3.284	0.148	0.948	1.315	0.123	0.952
Cpd (2)	1.704	2.141	0.919	3.03	0.132	0.942	2.869	0.117	0.981
Cpd (3)	0.023	0.615	0.965	1.429	0.117	0.982	0.478	0.22	0.992
Cpd (4)	0.268	0.514	0.978	2.158	0.144	0.98	0.84	0.166	0.996
Cpd (5)	1.165	0.49	0.901	0.638	0.067	0.991	0.914	0.26	0.972
Cpd (6)	0.745	0.495	0.972	1.374	0.061	0.997	0.35	0.265	0.991

Based on the Pseudo-second rate constants (k_2_ values) in Table 2**,** the kinetic adsorption rate of metal ions can be ranked a: (i) Cpd2 > Cpd1 > Cpd3 ≥ Cpd5 > Cpd6 > Cpd0 = Cpd1 for Cu^2+^ ion, (ii) Cpd1 > Cpd4 > Cpd2 > Cpd3 > Cpd5 > Cpd6 > Cpd0 for Ba^2+^ ion, and (iii) Cpd5 > Cpd6 > Cpd1 = Cpd2 = Cpd3 = Cpd4 > Cpd0 for Sr^2+^ ion. These data verified that the adsorption affinity of hydrogels for metal ions depends on both surface functionalities and the type of metal ions. From the Elovich model, the parameter α (mg/g.h) represents the number of ions adsorbed when the natural logarithm of (t)(lnt) is equal to zero, which corresponds to the adsorption quantity at the initial (t)(t = 1 h). Besides, the β values refer to the availability of adsorption sites on the prepared hydrogels for metal ions capture. By analyzing the importance of β and α, it is possible to understand the hydrogel's adsorption behavior and capacity for the studied metal ions. From Table 2, Cpd2 hydrogel demonstrated the highest β and α values of Cu^2+^ (2.141 g/mg and 1.704 mg/g.h, respectively), indicating its higher affinity for Cu^2+^ ions uptake compared with other chemically modified hydrogels. Besides, Cpd1 and Cpd0 recorded the maximum and minimum values of β for Ba^2+^ ions adsorption, recoding 0.148 and 0.033 g/mg, respectively. However, Cpd1 had the maximum α value of 3.284 mg/g.h for Ba^2+^ ions. Regarding Sr^2+^ adsorption, Cpd6 and Cpd2 recorded the highest β and α values of 0.265 g/mg and 01.315 mg/g.h, respectively. These variations in β and α values for six Cpdx (x= 1 to 6) samples can be ascribed to the influence of the adsorbent functionalities, the nature of the metal ions, and the experimental conditions on the adsorption process. For example, certain functional groups may exhibit stronger interactions with specific metal ions, resulting in higher adsorption capacities. The pore size distribution and overall porosity of the adsorbent material can also affect the accessibility and diffusion of metal ions into the material. Samples with more prominent pores or higher porosity may provide better access to the adsorption sites, resulting in higher β values. Conversely, samples with smaller pores or lower porosity may have restricted diffusion, leading to lower β values. Besides, the surface chemistry of the adsorbent can influence the electrostatic interactions and chemical reactions among the adsorbent and metal ions.

The above kinetic results highlight the influence of the modification strategies on the adsorption kinetics and effectiveness of hydrogel for different metal ions. This further indicates that the adsorption is primarily driven by van der Waals forces and electrostatic interactions among the adsorbent surface and the metal ions in the solution^37^. The sharing or trading of electrons among the hydrogel functionalities (–SO_3_^−^ and –COO^−^) and the HMs cations results in the involvement of the valent forces. The existence of additional exchangeable groups resulting from the chemical modification approach of the hydrogel likely enhances the adsorption performance, as evidenced by the higher removal rates observed for Cu^2+^ and Ba^2+^ ions compared to that obtained by the blank p(AAm*-co-*AAc) hydrogel (Cpd0) sample. This modification approach introduces more binding sites on the hydrogel surface, increasing the availability of active sites for metal ion adsorption by sharing or exchanging electrons among adsorbent and adsorbate ^38^.

#### Effect of adsorption temperature: thermodynamic study

**Figure 7:**
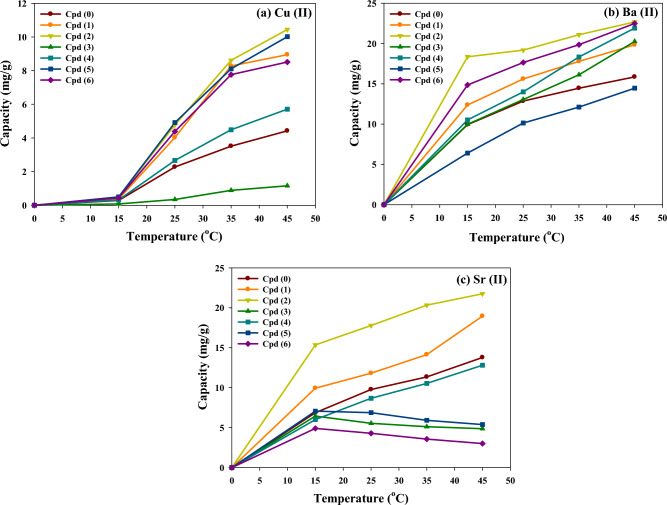
The impact of temperature on the adsorption capacity of three metal ions by p(AAm*-co-*AAc) hydrogels before and after functionalization processes.

Understanding the thermodynamic behavior can provide valuable insights into the energetics of the adsorption approach and help optimize conditions for efficient metal ion removal in practical applications. Figure 7 shows that the adsorption of Cu^2+^ and Ba^2+^ onto the modified and bare p(AAm*-co-*AAc) adsorbents is favored at higher temperatures. An increase in the temperature enhances the adsorption processes of Cu^2+^ and Ba^2+^, owing to their favorable endothermic adsorption. In this case, the higher temperature provides the necessary energy for adsorption and facilitates the interaction between metal ions and adsorbent surfaces. On the other hand, the adsorption of Sr^2+^ onto Cpd3, Cpd5, and Cpd6 is significantly declined as the temperature rises (i.e., an exothermic adsorption nature). This phenomenon can be attributed to the thermodynamic characteristics of the adsorption process, where the energy released during adsorption is sufficient to counteract the increased thermal energy at higher temperatures.

**Figure 8:**
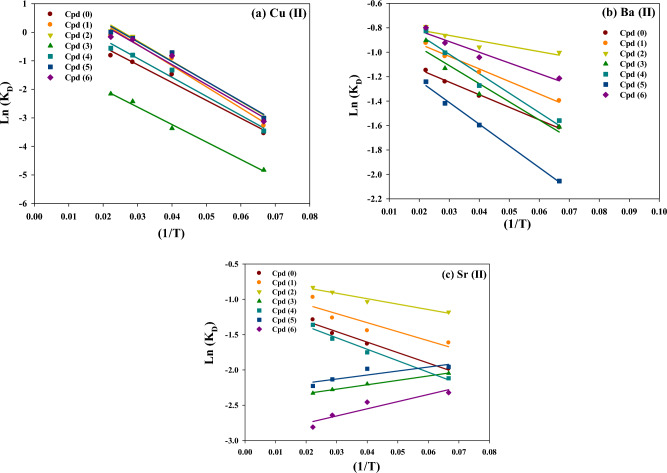
Graphical relationship plots between lnK_D_ and 1/T to determine the enthalpy change (∆H°) of (a) Cu^2+^, (b) Ba^2+^, and (c) Sr^2+^ ions adsorbed by the prepared hydrogel adsorbents.

To further understand the adsorption thermodynamics, the thermodynamic parameters of the three metal ions onto the prepared hydrogel adsorbent are calculated using the Eq. (9). The plot of lnK_D_ against 1/T will be used to calculate ΔH°, as shown in Fig. 8. It should be noted that a spontaneous system will display a decrease in ΔG° and ΔH° values with increasing temperature^39^.

9 $$ln{K}_{D}= \frac{\Delta S}{R}- \frac{\Delta H}{RT}$$

- lnK_D_: This represents the natural logarithm of the equilibrium constant. The equilibrium constant measures the extent to which a chemical reaction reaches equilibrium, indicating the ratio of products to reactants at equilibrium.
- ∆S: This term represents the change in entropy of the system. Entropy is a measure of the disorder or randomness of a system. A positive ∆S value suggests an increase in randomness, while a negative value suggests a decrease.
- R: The gas constant (0.08314 kJ/(mol·K)) is a fundamental thermodynamic constant.
- ∆H: This term represents the change in enthalpy of the system. Enthalpy measures the heat energy absorbed or released during a chemical reaction. A negative ∆H value suggests an exothermic reaction (heat released), while a positive value suggests an endothermic reaction (heat absorbed).
- T: The temperature of the system in Kelvin (K).

Table 3 presents the thermodynamic constants of the three adsorbed metal ions by blank and modified hydrogels based on Van’t Hoff plots in Fig. 8. As can be noticed, the positive ΔH° values for Cu^2+^, Ba^2+^, and Sr^2+^ metal ions adsorption indicate that the adsorption process is endothermic. An exception is the adsorption process of Sr^2+^ ions by Cpd3, Cpd5, and Cpd6, which recorded negative ΔH° values (i.e., exothermic adsorption process). Depending on the specific hydrogel samples, the positive and negative values of ΔH° for Sr^2+^ adsorption suggest both exothermic and endothermic processes. The endothermic adsorption verified the crucial role of temperature in enhancing metal ions' adsorption uptake, especially Cu^2+^, which needs higher activation energy to induce chelating/complex interactions with hydrogel surface functionalities. For Cu^2+^ adsorption, the ΔH° values ranged from 25.39 kJ/mol (Cpd0) to 92.88 kJ/mol(Cpd2), demonstrating the dominant chemical adsorption mechanism. However, for Ba^2+^/Sr^2+^ ions adsorption, the low adsorption enthalpy (ΔH° less than 60 kJ/mol) indicates that their adsorption behaviors are particular and involve physical and weak chemical adsorption interactions with hydrogel surface functionalities. The exothermic adsorption of Sr^2+^ ions by Cpd3, Cpd5, and Cpd6 indicates stronger adsorbate-adsorbent interactions with heat released during the adsorption process. In contrast, the enhanced adsorption of metal ions with temperature could be ascribed to the improved mass transfer/diffusivity rate through the hydrogel porous networks. The variation in ΔH° values across different samples highlights the influence of the adsorbent composition on the adsorption behavior of each metal ion. These thermodynamic parameters provide insights into the energetics of the adsorption process, indicating the strength and nature of the interaction between the metal ions and the adsorbent. The positive ΔS° values for metal ions also indicate the rise in randomness at the liquid–solid interface during adsorption. This randomness increases with increased metal ion activity in water and their interactions with adsorbent surface sites. The variation in the ΔG° (negative and positive values) also indicates the thermodynamic feasibility of the adsorption process, and the adsorption interaction is less favorable at lower temperatures. These findings highlight the importance of considering the thermodynamic aspects of the adsorption process, particularly the exothermic or endothermic nature, when studying the adsorption of different metal ions onto specific adsorbents.

**Table 3:** The thermodynamic parameters (ΔH°, ΔG°, and ΔS°)* of the three adsorbed metal ions onto the blank and modified hydrogel adsorbents.

Hydrogels/Constants*	Cu^2+^			Ba^2+^			Sr^2+^		
	ΔH°	ΔS°	ΔG°	ΔH°	ΔS°	ΔG°	ΔH°	ΔS°	ΔG°
Cpd (0)	25.39	0.08	2.16	24.17	0.08	1.57	28.91	0.09	2.82
Cpd (1)	80.37	0.28	− 1.78	34.25	0.11	0.46	38.14	0.12	1.99
Cpd (2)	92.88	0.33	− 5.85	32.42	0.11	− 1.24	37.17	0.13	− 0.52
Cpd (3)	62.55	0.18	8.16	46.17	0.15	1.50	− 9.12	− 0.05	4.83
Cpd (4)	32.01	0.10	1.39	57.68	0.19	1.12	29.87	0.09	3.28
Cpd (5)	55.47	0.22	− 8.19	34.13	0.10	2.67	− 9.70	− 0.05	4.12
Cpd (6)	56.03	0.20	− 3.15	44.80	0.15	− 0.45	− 14.86	− 0.07	5.62

*Thermodynamic constants: Standard enthalpy change (ΔH: kJ/mol), Free energy change (ΔG: kJ/mol), and Entropy change (ΔS: kJ/mol.K).

The above thermodynamic data are further verified by the calculated activation energies (Ea) for the adsorption of Cu^2+^, Ba^2+^, and Sr^2+^ ions by the modified p(AAm-co-AAc) hydrogels using the Arrhenius equation: k = A e^(−Ea/RT)^, where k is rate constant, A is pre-exponential factor, Ea is activation energy (kJ/mol) by taking the natural log of the Arrhenius equation. ln(k) = ln(A) − (Ea/R)(1/T).A plot of ln(k) vs 1/T will give a straight line with slope = − Ea/R. From this slope, the activation energy Ea can be calculated and summarized in Table 4. These values represent the energy required for the adsorption process to take place. Lower activation energies indicate that adsorption is more favorable and occurs more quickly. Conversely, higher activation energies suggest that adsorption is less favorable and requires more energy. The specific values provide insights into the adsorption behavior of these metal ions on the different hydrogel samples. For instance, in most cases, Cu^2+^ ions show higher activation energies, suggesting a higher energy barrier for their adsorption. Ba^2+^ ions exhibit relatively lower activation energies, making them easier to adsorb.

**Table 4:** The activation energies (Ea: kJ/mol) for the adsorption of Cu^2+^, Ba^2+^, and Sr^2+^ ions by the blank and modified p(AAm-co-AAc) hydrogel adsorbents.

Sample	Cu^2+^ Ea (kJ/mol)	Ba^2+^ Ea (kJ/mol)	Sr^2+^ Ea (kJ/mol)
Cpd (0)	59.7	53.2	56.1
Cpd (1)	63.2	57.6	58.9
Cpd (2)	71.5	50.8	55.6
Cpd (3)	58.4	55.1	61.7
Cpd (4)	60.9	59.6	59.2
Cpd (5)	64.8	56.3	63.5
Cpd (6)	64.1	49.5	57.8

### Comparative study

Table 5 shows the comparative adsorption capacities of different adsorbents for removing Cu^2+^, Ba^2+^ and Sr^2+^ from water. Modified fly ash and graphene oxide sheets showed Cu^2+^ removal capacities of 53.5 mg/gand 46.6 mg/g, respectively. Polyethylene glycol diacrylate-3-sulfopropyl methacrylate potassium salt exhibited the highest Cu adsorption capacity of 99 mg/g. Magnetic chitosan beads and hydroxyapatite were effective for Sr^2+^ removal with capacities of 11.58 mg/gand 28.51 mg/g, respectively. itanium silicate and TiY2O5@g-C3N4 had high Ba^2+^ adsorption of 144 mg/g and 295 mg/g. Allophane showed 38.6 mg/g and 34.4 mg/g capacities for Ba^2^ and Sr^2^, respectively. P(CTS-AAm) and Chitosan-g-maleic acid had excellent Cu removal of 196.8 mg/g and 312.4 mg/g. The present workshowed relatively lower but significant capacities of 13.67 mg/g 36.4 mg/g and 27.31 mg/g for Cu^2^, Ba^2^and Sr^2^ removal in 1000 ppm of NaCl solution. Thus different low-cost adsorbents can be effectively used for heavy metal remediation from water.

**Table 5:** Comparative of different adsorbents for adsorption removal of Cu^2+^, Ba^2+^, and  ^2^ Sr^2+^ ions from water solution.

Adsorbent	Cu ^2+^ (mg/g)	Ba^2+^ (mg/g)	Sr^2+^ (mg/g)	Ref
Modified fly ash	53.5	N/A	N/A	^40^
Modfied graphene oxide (GO) sheets	46.6	N/A	N/A	^41^
Polyethylene glycol diacrylate-3-sulfopropyl methacrylate potassium salt (PEGDA-SMP)	99	N/A	N/A	^42^
Magnetic chitosan beads	N/A	N/A	11.58	^43^
Alkali activated metakaolin	N/A	N/A	167.5	^44^
Hydroxyapatite	N/A	N/A	28.51	^45^
Titanium silicate	N/A	144	N/A	^46^
TiY_2_O_5_@g-C_3_N_4_	N/A	295	N/A	^47^
Allophane	N/A	38.6	34.4	^48^
P(CTS-AAm)	196.8	N/A	N/A	^49^
Chitosan-g-maleic acid	312.4	N/A	N/A	^50^
Precent wor	13.67	36.4	27.31	In 1000 PPm of NaC

## Conclusions

The radiation synthesis of p(AAm*-co-*AAc) adsorbents is based on chemically developed for the removal of three metal ions (Cu^2+^, Ba^2+^, and Sr^2+^) from a synthetic saline solution (NaCl 1000 ppm) is successively performance. The observed trend suggests that co-monomer concentration can be optimized to achieve the desired application swelling properties. Lower co-monomer concentrations can be preferred when high swelling capacity is required, while higher concentrations can be suitable for a more compact and less-swelling hydrogel. The swelling degrees of the prepared p(AAm*-co-*AAc) hydrogels are lower when the acrylic acid content is higher than those with higher polyacrylamide content. The lower swelling degrees of hydrogels with higher acrylic acid content can be attributed to intermolecular hydrogen bonds formed by acrylic acid molecules, which restrict the uptake of water molecules by the hydrogel, leading to a decrease in swelling capacity. Further investigation and optimization of irradiation dose can help tailor hydrogel properties for specific applications requiring controlled swelling behavior and mechanical strength. Variations in pore diameter and hydrogel wall thickness are observed in the modified samples. Hydrogels with stronger hydrogen bonding exhibit smaller pore sizes, while those with weaker hydrogen bonding show larger pore sizes. During the adsorption process, it was also found that the adsorption performance of p(AAm*-co-*AAc) hydrogel depends mainly on the modifying agent and the specific HM ion being targeted (e.g., Cu^2+^, Ba^2+^, and Sr^2+^). Adsorption results showed that the chemically modified hydrogel with sodium hydroxide (Cpd 2) exhibited the highest affinity for the adsorption recovery of the three HM ions. Based on adsorption isotherm, kinetic, and thermodynamic studies, the adsorption process of Cu^2+^, Ba^2+^, and Sr^2+^ ions onto the modified p(AAm*-co-*AAc) hydrogel samples followed a favorable multilayer adsorption mechanism, especially at higher temperature. The adsorption energy also indicates the dominance of chemical interaction between Cu ions and hydrogel surface functionalities, while the adsorption of Ba^2+^and Sr^2+^ions involves moderate to strong adsorption interactions, including a complex physicochemical adsorption phenomenon. These adsorption data can contribute to further understanding the suitability and effectiveness of these hydrogels as adsorbents for metal ion removal in various applications (Supplementary Information).

### Supplementary Information

Supplementary Information.

## Data Availability

The data that support the findings of this study are available from the corresponding author upon reasonable request.
