# Supplementary material for: Radiation synthesis and chemical modifications of p(AAm-co-AAc) hydrogel for improving their adsorptive removal of metal ions from polluted water
Source: Sci Rep. 2023 Dec 11;13:21879. doi: 10.1038/s41598-023-49009-0 (PMC10710999; doi:10.1038/s41598-023-49009-0)
Supplement: Supplementary file 1 — Supplementary Information. [file 41598_2023_49009_MOESM1_ESM.docx]

**Electronic supplementary information (ESI) file**

**Radiation synthesis and chemical modifications of p(AAm*-co-*AAc) hydrogel for improving their adsorptive removal of metal ions from polluted water**

**Israa kamal Abdel Maksoud^1^, Ghada Bassioni^2^, Norhan Nady^3^, Sherif A. Younis^4*^, Mohamed Mohamady Ghobashy^5*^, and M. S. A. Abdel-Mottaleb^1^**

^1^Department of Chemistry, Faculty of Science, Ain Shams University, Abbassia, Cairo, 11566 Cairo, Egypt; ^2^Department of Physics and Engineering Mathematics, Faculty of Engineering, Ain Shams University; ^3^Polymeric Materials Research Department, City of Scientific Research and Technological Applications (SRTA-City), New Borg El-Arab City, Alexandria, 21934, Egypt. ^4^Analysis and Evaluation Department, Egyptian Petroleum Research Institute (EPRI), Nasr City, Cairo 11727, Egypt (sherifali_r@yahoo.com); ^5^Radiation research of Polymer Department. National Center for Radiation Research and Technology (NCRRT), Egyptian Atomic Energy Authority (EAEA).[Mohamed.ghobashy@eaea.org.eg](mailto:Mohamed.ghobashy@eaea.org.eg)

**S1. Adsorption isotherm models**

1. **Freundlich isotherm model**

The Freundlich isotherm is a widely used equilibrium isotherm model but provides no information on the monolayer adsorption capacity, in contrast to the Langmuir model. The Freundlich isotherm model assumes neither homogeneous site energies nor limited adsorption levels.

1. **Langmuir isotherm model**

It's significant to note that the Langmuir equation assumes ideal conditions of monolayer adsorption and may not be applicable if the assumptions are unmet or the adsorption approach involves multilayer adsorption or interactions among adsorbed molecules.

1. **Tempkin isotherm model**

Tempkin isotherm considered the effects of indirect adsorbent/adsorbate interactions on the adsorption process. The Tempkin isotherm assumes a uniform distribution of adsorption heat over the surface of the adsorbent and considers the influence of adsorbent-adsorbate interactions on the adsorption process. It suggests that the heat of adsorption decreases linearly with increasing coverage, indicating that the adsorbate-adsorbent interaction weakens as more adsorbate molecules are adsorbed. The Tempkin isotherm can provide insights into the adsorption behavior and the strength of adsorbate-adsorbent interactions. However, it is significant to note that the Tempkin isotherm assumes certain ideal conditions and may not be applicable in all cases. Other factors, like the specific characteristics of the adsorbent, the adsorbate, and the experimental conditions, should be considered when interpreting the results obtained from the Tempkin isotherm.

**S2. Adsorption kinetic models**

The pseudo-first-order model assumes that the adsorption rate is directly proportional to the difference between the equilibrium adsorption capacity and the adsorbate concentration at any given (t). This model provides a simple exponential decay equation and estimates the rate constant for the adsorption process. It is widely used for its simplicity and ease of application. On the other hand, the pseudo-second order model assumes that the adsorption rate is proportional to the square of the remaining concentration of the adsorbate. This model suggests a chemisorption mechanism or chemical interactions between the adsorbent surface and the adsorbate. It provides a more accurate description of adsorption kinetics and is commonly used to determine the equilibrium adsorption capacity and the rate constant associated with the second-order process. The Elovich model is often employed to analyze the initial stages of adsorption, especially in cases where chemisorption is the dominant mechanism. This model considers the surface coverage and the initial adsorption rate as parameters.
